# Pharmacokinetics and tolerability of cabotegravir and rilpivirine long-acting intramuscular injections to the *vastus lateralis* (lateral thigh) muscles of healthy adult participants

**DOI:** 10.1128/aac.00781-23

**Published:** 2023-12-01

**Authors:** Kelong Han, Hakop Gevorkyan, Jafar Sadik Shaik, Herta Crauwels, Claudia Leemereise, Gilda Bontempo, Beta Win, Vasiliki Chounta, Ciara Seal, Rebecca DeMoor, Ronald D'Amico, William R. Spreen, Susan L. Ford

**Affiliations:** 1 Department of Clinical Pharmacology, Modeling & Simulation, GSK, Collegeville, Pennsylvania, USA; 2 California Clinical Trials Medical Group in affiliation with PAREXEL, Glendale, California, USA; 3 Department of Clinical Pharmacology & Pharmacometrics, Janssen Research & Development, Spring House, Pennsylvania, USA; 4 Department of Clinical Pharmacology, Janssen Research & Development, Beerse, Belgium; 5 GSK, Amersfoort, the Netherlands; 6 ViiV Healthcare, Durham, North Carolina, USA; 7 GSK, Stevenage, United Kingdom; 8 ViiV Healthcare, Brentford, United Kingdom; 9 GSK, Collegeville, Pennsylvania, USA; 10 Department of Clinical Pharmacology, Modeling & Simulation, GSK, Durham, North Carolina, USA; Providence Portland Medical Center, Portland, Oregon, USA

**Keywords:** cabotegravir, rilpivirine, thigh, long-acting, pharmacokinetics, HIV-1

## Abstract

Cabotegravir + rilpivirine administered via intramuscular gluteal injections is the first complete long-acting (LA) regimen approved for maintaining HIV-1 virologic suppression. The *vastus lateralis* (lateral) thigh muscle could be a potential alternative site of administration in circumstances such as injection site fatigue, intolerability, or contraindication for gluteal administration. Cabotegravir and rilpivirine pharmacokinetics and participant tolerability were evaluated following single intramuscular injections to the lateral thigh. Healthy adult participants received 4 weeks of daily oral cabotegravir (30 mg) and rilpivirine (25 mg), followed by a 10- to 14-day washout and single 3 mL intramuscular injections of cabotegravir LA 600 mg and rilpivirine LA 900 mg to the lateral thigh. Safety, tolerability, and pharmacokinetics were evaluated through 52 weeks post injection. Pharmacokinetic parameters were estimated using non-compartmental analysis. Fifteen participants (female at birth, *n* = 6) enrolled. Median age was 33 years. Median weight was 93.6 kg. Median body mass index was 31.4 kg/m^2^. One participant withdrew due to pregnancy after oral dosing before receiving an injection. Plasma concentrations at Weeks 4 and 8 were 15.4- and 5.3-fold above the protein-adjusted 90% inhibitory concentration for cabotegravir and 4.7- and 2.4-fold for rilpivirine, respectively. The most common injection site reactions were pain [28/28 (100%)], induration [15/28 (54%)], and swelling [12/28 (42%)]; 94% were Grade 1 or 2. Cabotegravir and rilpivirine plasma pharmacokinetic profiles observed in this study support further evaluation of thigh administration in target populations of people living with HIV-1. Tolerability of cabotegravir + rilpivirine LA intramuscular lateral thigh injections was similar to gluteal administration.

## INTRODUCTION

Cabotegravir (CAB), an integrase strand transfer inhibitor, plus rilpivirine (RPV), a non-nucleoside reverse transcriptase inhibitor, is the first approved complete long-acting (LA) regimen recommended by treatment guidelines for the maintenance of HIV-1 virologic suppression (1, 2). CAB + RPV LA may address some of the challenges associated with daily oral antiretroviral therapy, such as privacy concerns, challenges and anxiety related to maintaining adherence, and the daily reminder of HIV status (3). In Phase 3/3b studies, CAB + RPV LA intramuscular (IM) gluteal injections into the ventrogluteal [recommended injection site (4)] or dorsogluteal muscle demonstrated noninferiority to daily oral therapy when administered every 4 weeks (Q4W) in the FLAIR and ATLAS studies (5, 6), as well as when administered every 8 weeks (Q8W) compared with Q4W dosing in the ATLAS-2M study (7).

CAB and RPV pharmacokinetic (PK) data following IM gluteal injections have been reported previously for both CAB LA and RPV LA, individually and in combination (CAB + RPV LA), in people with and without HIV-1 (6 – 13). Population PK analyses have further characterized the PK of CAB LA and RPV LA (14, 15). CAB LA and RPV LA exhibit absorption-limited (flip-flop) kinetics, with a terminal half-life of 25–54 days for CAB LA (12, 13) and approximately 200 days for RPV LA (15). Phase 2/3/3b studies investigating CAB + RPV LA dosed Q4W or Q8W as a combination regimen in people living with HIV-1 demonstrated median plasma CAB LA and RPV LA IM trough concentrations that remained well above (approximately >8-fold for CAB and >3-fold for RPV) their respective protein-adjusted 90% inhibitory concentrations (PA-IC_90_: CAB, 0.166 µg/mL; RPV, 12.0 ng/mL) throughout assessment periods up to 152 weeks on CAB + RPV LA treatment (6, 7, 11, 16, 17).

The *vastus lateralis* (lateral thigh) muscle could be a potential alternative site of IM administration, helping to alleviate injection site fatigue or intolerability. Alternative IM injection sites could also be useful in instances of inaccessibility of the gluteal muscle (e.g., buttock implants or insufficient gluteal mass) or intermittently when gluteal injection is not ideal (e.g., prior to prolonged sitting). The *vastus lateralis* has been used as an injection location for the administration of other drugs, including epinephrine and vaccines, and is a common injection site in children (18, 19).

Here, we present the results of a Phase 1 study (NCT04371380) evaluating the PK and tolerability following single injections of CAB LA and RPV LA administered separately to contralateral thigh muscles of adults without HIV infection.

## MATERIALS AND METHODS

### Study design and participants

This Phase 1, open-label study assessed the PK and tolerability of CAB LA and RPV LA single-dose IM injections in healthy adults. Eligible participants were between 18 and 50 years of age and overtly healthy, as determined by medical evaluation including medical history, physical examination, laboratory tests, and cardiac monitoring, with a body mass index (BMI) within the range of 18–35 kg/m^2^. There was no formal calculation of power or sample size for this study; a target of approximately 15 participants (≥30% female) was considered sufficient to provide informative PK and safety data following CAB LA + RPV LA IM injections to the lateral thigh. Female participants were eligible to participate if not pregnant, breastfeeding, or of child-bearing potential, or if they were using highly effective birth control methods (<1% failure). Contraceptive use (including hormonal contraceptives) was consistent with local regulations for those participating in clinical studies. Participants were negative on two consecutive tests for SARS-CoV-2, performed at screening and on Day –1 of admission to the Phase 1 unit.

**Fig 1:**
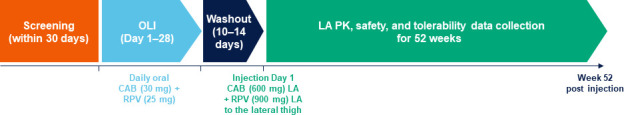
Study design. PK sampling times for CAB and RPV concentrations at pre-injection, 1 hour and 2 hours post injection, on Days 2, 4, 5, 7/8, 10, 15, 17, and 22 postinjection, and at Weeks 4 (Day 28), 8, 12, 24, 36, and 52, and at withdrawal visit. CAB, cabotegravir; LA, long-acting; OLI, oral lead-in; PK, pharmacokinetics; RPV, rilpivirine.

After a 30-day screening period, eligible participants received oral CAB (30 mg) and RPV (25 mg) once daily for 28 days as an oral lead-in (OLI) tolerability assessment. Following a 10- to 14-day washout period upon conclusion of the OLI, participants received single separate CAB LA and RPV LA injections, administered intramuscularly: 600 mg (1 × 3 mL) CAB LA injection to the left *vastus lateralis* muscle and 900 mg (1 × 3 mL) RPV LA injection to the right *vastus lateralis* muscle (Fig. 1).

This study was conducted at Parexel Los Angeles in accordance with the Declaration of Helsinki and Council for International Organizations of Medical Sciences’ International Ethical Guidelines. Written informed consent was obtained from all participants. The study protocol, amendments, informed consent, and other information were reviewed and approved by Institutional Review Board Services at Aspire Independent Review Board, Santee, California, USA. The trial is registered as NCT04371380 at ClinicalTrials.gov.

### Objectives and endpoints

The primary objective of the study was to describe the PK profiles of CAB LA and RPV LA following single IM injections of each to the lateral thigh muscle in healthy adult participants. Specifically, PK endpoints assessed were as follows: maximum observed concentration (C_max_) in plasma; time of maximum observed concentration (T_max_); concentration at Weeks 4 and 8; area under the concentration–time curve (AUC) from time zero to last quantifiable time point (AUC_last_) through the follow-up phase; AUC from time zero to infinity (AUC_0–∞_); terminal phase half-life (t_½_); and LA absorption rate constant (KA_LA_).

The safety and tolerability of CAB and RPV following repeated oral doses and single IM injections in the lateral thigh were also assessed. Safety and tolerability parameters included clinical laboratory tests, electrocardiograms (ECGs), vital sign assessments, and adverse events (AEs), including injection site reactions (ISRs). Exploratory endpoints included the assessment of post injection pain and the acceptance of pain and ISRs following IM thigh injections.

### Procedures

#### PK and statistical analysis

Safety, patient-reported tolerability and acceptability, and PK data were collected through 52 weeks post injection. Two 2 mL blood samples—one in K_3_EDTA for CAB and one in sodium heparin for RPV – were collected at pre-injection, 1 hour and 2 hours post injection, on Days 2, 4, 5, 7/8, 10, 15, 17, and 22 post injection, at Weeks 4 (Day 28), 8, 12, 24, 36, and 52, and at withdrawal visits for the determination of plasma PK concentrations. Blood samples were protected from light throughout processing due to RPV light sensitivity. Following centrifugation of blood samples at ~2,500–3,000 rpm for 10 minutes, plasma was transferred by pipette to amber/brown polypropylene tubes (Sarstedt, Nümbrecht, Germany) and stored at –20°C, pending analysis. CAB was extracted from 25 µL of human plasma by protein precipitation using 50:50 acetonitrile:water containing ^13^C^2^H_2_^15^N-CAB as an internal standard; and RPV was extracted from 100 µL of human plasma using acetonitrile containing ^13^C-D_4_-RPV as an internal standard. CAB and RPV extracts were analyzed by high-performance liquid chromatography–mass spectrometry using a Turbo-IonSpray interface and multiple reaction monitoring. The analytical linear ranges were 25–25,000 ng/mL (0.025–25 µg/mL) for CAB and 1–500 ng/mL for RPV.

Safety assessments were made at each visit. Although required to use highly effective contraceptive methods (<1% failure), women of child-bearing potential underwent testing for pregnancy (urine or serum as required by local regulations) within 30 days of the first dose and at regular intervals during both the OLI and injection phases of the study. Serum pregnancy testing was required following any equivocal urine test result.

Participant-reported maximum level of pain following injections was assessed by the numerical rating scale (NRS), ranking pain from 0 “no pain” to 10 “extreme pain” at Days 1, 2, 4, 5, and 7/8. The acceptance of pain and ISRs was assessed using the “acceptability of ISR” domain of the perception of injection (PIN) questionnaire, ranking acceptance of injections from 1 “totally acceptable” to 5 “not at all acceptable” at Day 8.

Plasma PK parameters were estimated using non-compartmental analysis with linear-up/log-down methods. KA_LA_ was assumed to be equal to lambda-z. Estimations of AUC_0–∞_, t_½_, and KA_LA_ were deemed valid and reportable when all of the following three criteria were met: the time span used for the linear regression line (LAMZ SPAN) was greater than twice the t_½_; the percentage of AUC_0–∞_ obtained by extrapolation (%AUC_extra_) was <20% of AUC_0–∞_; and the R-squared value of the linear regression was >0.85. PK parameters were summarized using descriptive statistics. No formal statistical hypotheses were tested. Where appropriate, an estimation approach was taken, and point estimates and confidence intervals were constructed.

## RESULTS

### Disposition and participant characteristics

In total, 21 individuals were screened and 15 enrolled in the study; three did not meet the inclusion/exclusion criteria, two individuals withdrew consent, and one individual was lost to follow-up during screening. Of the 15 participants who enrolled, six (40%) were female (sex at birth), seven (47%) were White, and the median (range) age was 33 (21–49) years; the median (range) weight was 93.6 (67.9–107.7) kg and the median (range) BMI was 31.4 (24.3–34.4) kg/m^2^ (Table 1). All 15 participants received study drugs in the OLI phase and 14 participants received study drugs in the IM injection phase. One participant tested positive for pregnancy using urine and serum human chorionic gonadotropin between the OLI and injection phase tests and was withdrawn at the investigator’s discretion on Day 86 without receiving an injection. Of the 14 participants receiving CAB LA and RPV LA injections, one [female (sex at birth), BMI = 34.4 kg/m^2^] had a leakage of approximately 0.5 mL (~17% of 3 mL dose) of CAB from the injection site and was excluded from summaries of CAB PK parameters except for t_½_ and KA_LA_; an additional two participants were not included in descriptive statistics of Week 4 CAB and RPV concentrations due to out-of-window Week 4 visits.

**TABLE 1:** Baseline characteristics^*b*^

Parameter	CAB + RPV (*N* = 15)^*a*^
Median age (range), years	33 (21–49)
Female (sex at birth), *n* (%)	6 (40)
Male (sex at birth), *n* (%)	9 (60)
Race, *n* (%)	
White	7 (47)
Black or African American	7 (47)
Asian	1(7)
Hispanic/Latinx, *n* (%)	5 (33)
Median BMI (range), kg/m^2^	31.40 (24.3–34.4)
Median weight (range), kg	93.6 (67.9–107.7)

^*a*^ One participant withdrew prior to receiving an injection.

^*b*^ BMI, body mass index; CAB, cabotegravir; RPV, rilpivirine.

### PK parameters

**Fig 2:**
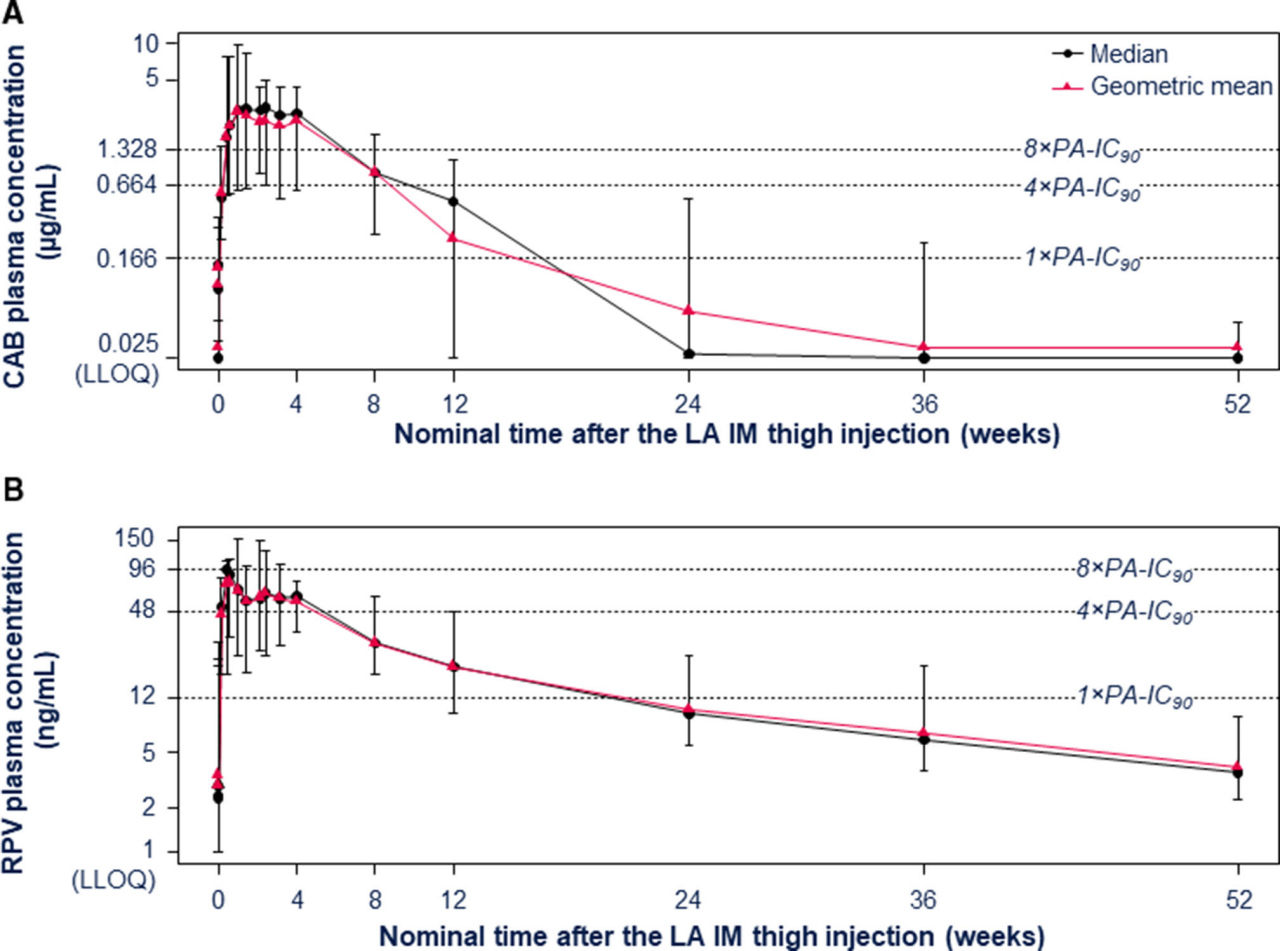
Plasma concentration–time profiles of CAB (A) and RPV (B). Error bars represent minimum and maximum observed concentrations. Non-quantifiable concentrations were imputed as LLOQ for the purpose of calculating statistics. CAB, cabotegravir; IM, intramuscular; LA, long-acting; LLOQ, lower limit of quantitation; PA-IC_90_, protein-adjusted 90% inhibitory concentration; RPV, rilpivirine.

Plasma concentrations of CAB LA following IM thigh injection (*n* = 13) showed a geometric mean C_max_ of 3.38 µg/mL, with a median T_max_ of 166 hours (7 days). The geometric mean AUC_last_ was 3,612 hours × μg/mL and the geometric mean AUC_0–∞_ was 3,948 hours × μg/mL. The geometric mean t_½_ was 21 days, and the geometric mean KA_LA_ was 0.0014 /h (Table 2). The geometric mean CAB plasma concentration following a single IM dose at Week 4 was 2.56 µg/mL. Geometric mean CAB plasma concentrations were 15.4- and 5.3-fold above the PA-IC_90_ (0.166 µg/mL) at Weeks 4 and 8, respectively, and remained above the PA-IC_90_ until at least Week 12 (Fig. 2A).

**TABLE 2:** Plasma PK parameters of CAB and RPV after IM administration in the lateral thigh^*b*^ ^,^ ^*c*^

	C_max_	T_max_	AUC_Iast_	AUC_(0–Week 12)_	AUC_0–∞_	t_½_	KA_LA_	Concentration at Week 4
CAB LA (*n* = 13)	3.38 µg/mL (66.0) [2.35, 4.86] (1.02, 9.60)	7 days (7, 55)	3,612 h × μg/mL (23.0) [3,149, 4,142] (2,488, 5,108)	3,202 h × μg/mL (32.8) [2,582, 3,969] (1,629, 4,490)	3948 h × μg/mL (21.5) [3,391, 4,596] (2,509, 5,168)	21 days (79.3) [13.0, 35.2] (8.87, 60.2)	0.0014 /h (79.3) [0.0008, 0.0022] (0.0005, 0.0032)	2.56 µg/mL (38.9) [1.99, 3.30] (1.17, 4.39)
RPV LA^*a*^ (*n* = 14)	93.5 ng/mL (37.7) [75.8, 115] (35.4, 155)	5 days (3, 27)	143,891 h × ng/mL (33.0) [119,540, 173,202] (84,137, 283,228)	83,777 h × ng/mL (33.7) [69,313, 101,260] (47,374, 147,985)	171,953 h x ng/mL (31.1) [140,201, 210,896] (110,053, 314,053)^*a*^	137 days (26.6) [115, 163] (86, 194)^*a*^	0.000211 /h (26.6) [0.000177, 0.000252] (0.000149, 0.000335)^*a*^	56.7 ng/mL (28.5) [47.5, 67.7] (34.9, 79.0)

^*a*^ It should be noted that the 52-week sample time frame was too short to accurately determine the AUC_0–∞_, t_½_, and KA_LA_ for RPV LA. *N* = 11 for these parameters.

^*b*^ Values are displayed as geometric mean (CVb%) [95% CI] (minimum, maximum), except for T_max_, which is displayed as median days (minimum, maximum). Plasma concentrations below the lower limit of quantitation were omitted for estimating PK parameters.

^*c*^ AUC, area under the concentration–time curve; AUC_0–∞_, area under the concentration–time curve from time zero to infinity; AUC_last_, area under the concentration–time curve from time zero to last quantifiable time point; CAB, cabotegravir; CI, confidence interval; C_max_, maximum plasma concentration; CVb, coefficient of variation; IM, intramuscular; KA_LA_, absorption rate constant; LA, long-acting; NA, not available; PK, pharmacokinetics; RPV, rilpivirine; t_½_, terminal phase half-life; T_max_, time of maximum observed concentration.

Following RPV LA IM thigh injection (*n* = 14), the geometric mean C_max_ was 93.5 ng/mL, with a median T_max_ of 131 hours (5 days). The geometric mean AUC_last_ was 143,891 h × ng/mL and the geometric mean AUC_0–∞_ was 171,953 h × ng/mL. The geometric mean t_½_ was 137 days, and the geometric mean KA_LA_ was 0.000211 /h (Table 2). It should be noted that the 52-week sample time frame was too short to accurately determine the AUC_0–∞_, t_½_, and KA_LA_ for RPV. The geometric mean RPV plasma concentration following a single IM dose at Week 4 was 56.7 ng/mL. Geometric mean RPV plasma concentrations were 4.7- and 2.4-fold above the PA-IC_90_ (12 ng/mL) at Weeks 4 and 8, respectively, and remained above the PA-IC_90_ until at least Week 12 (Fig. 2B).

### Safety overview

No serious AEs or AEs leading to discontinuation were reported. All participants (100%, *n* = 15/15) reported at least one AE during the study. In total, 93% (*n* = 14/15) of participants reported AEs that were considered related to study drugs (Table 3). Excluding ISRs, drug-related AEs were chills (*n* = 3), headache, feeling hot, musculoskeletal stiffness, and insomnia (all *n* = 1); all were Grade 1 or 2.

**TABLE 3:** Safety overview^*c*^

Parameter, *n* (%)	CAB + RPV (*N* = 15)^*a*^
Any AE Excluding ISRs	15 (100) 9 (60)
Any drug-related AE Excluding ISRs	14 (93) 3 (20)
Grade ≥ 3 AE^*b*^ Excluding ISRs	3 (20) 0
Serious AEs	0
AEs leading to withdrawal	0

^*a*^ One participant withdrew prior to receiving an injection.

^*b*^ No Grade 4 or 5 AEs occurred. All Grade 3 ISRs were injection site pain.

^*c*^ AE, adverse event; CAB, cabotegravir; ISR, injection site reaction; RPV, rilpivirine.

ISRs were the most frequently reported AEs related to study drugs and were reported in all 14 participants who received an injection (Table S1). Of these, 5/14 (36%) had maximum Grade 1 ISRs, 6/14 (43%) had maximum Grade 2 ISRs, and 3/14 (21%) had maximum Grade 3 ISRs. At the event level, 81 ISRs were reported from 28 injections across the 14 participants who received an injection (Table 4). Most ISRs were Grade 1 (79%, *n* = 64/81) or 2 (15%, *n* = 12/81), with a median (interquartile range) duration of 8 (7–11) days. All Grade 3 ISRs were injection site pain, with no Grade 4 or 5 ISRs reported. ISR frequency, type, and severity were generally comparable by drug (CAB/RPV).

**TABLE 4:** ISR event-level safety overview^*b*^

Parameter	CAB + RPV LA (*N* = 15)	Drug	
		CAB (*N* = 15)	RPV (*N* = 15)
Participants who received ≥1 injection, *n* (%)	14 (93)	14 (93)	14 (93)
Number of injections	28	28	28
ISR events, *n*	81	44	37
Injection site pain, *n* (% of injections)	28 (100)	14 (50)	14 (50)
Injection site induration, *n* (% of injections)	15 (54)	8 (29)	7 (25)
Injection site swelling, *n* (% of injections)	12 (43)	7 (25)	5 (18)
Injection site erythema, *n* (% of injections)	11 (39)	6 (21)	5 (18)
Injection site bruising, *n* (% of injections)	6 (21)	4 (14)	2 (7)
Injection site warmth, *n* (% of injections)	5 (18)	3 (11)	2 (7)
Injection site pruritus, *n* (% of injections)	4 (14)	2 (7)	2 (7)
Grade 3 ISR events, *n* (% of ISRs)^*a*^	5 (6)	3 (7)	2 (5)
Median duration (IQR), days	8 (7–11)	8 (7–12)	8 (5–11)
Withdrawal due to injection-related reasons	0	0	0

^*a*^ No Grade 4 or 5 AEs occurred.

^*b*^ AE, adverse event; CAB, cabotegravir; IQR, interquartile range; ISR, injection site reaction; LA, long acting; RPV, rilpivirine.

There were no clinically meaningful changes from baseline in chemistry and hematology parameters. No changes in vital signs were reported as an AE and there were no obvious patterns detected across treatment groups or study visits. There were no ECG abnormalities that were considered clinically significant by the study investigator.

#### Pregnancy cases

One participant was withdrawn for pregnancy following completion of the OLI phase (at which time CAB and RPV trough concentrations were 47.2- and 9.7-fold above the PA-IC_90_ for CAB and RPV at 7.83 µg/mL and 116 ng/mL, respectively). The participant underwent elective termination of pregnancy 41 days after the reported last menstrual period (LMP) and 19 days after the last oral dose, with no apparent evidence of any congenital anomaly.

A second participant became pregnant ~30 weeks after receiving their CAB LA and RPV LA injections (estimated by reported LMP) (Fig. S1). CAB concentrations were below PA-IC_90_ (0.166 µg/mL) from ~6 weeks post LMP and non-quantifiable at Week 52; RPV concentrations were below PA-IC_90_ (12 ng/mL) throughout. This pregnancy resulted in a normal live birth at ~33 weeks gestation, 64 weeks after receiving their IM injections, and 12 weeks after the final study visit at Week 52.

### Patient-reported tolerability and acceptability

Mean post injection pain by NRS score reached a maximum at Day 2 and decreased by Day 8 for both CAB LA and RPV LA (Fig. S2).

Mean scores were ≤2.61 across all domains (1 “totally acceptable” to 5 “not at all acceptable”) and individual items of the PIN questionnaire for CAB LA and RPV LA (Fig. S3 and S4). Mean [standard deviation (SD)] PIN scores at Day 8 post injection were moderate and similar for both drugs across domains. At Day 8, acceptance of local reactions was rated as “totally acceptable” or “very acceptable” by 71% (*n* = 10/14) of participants for the CAB LA injection and 64% (*n* = 9/14) for the RPV LA injection. At Day 8, acceptance of pain was rated as “totally acceptable” or “very acceptable” by 29% (*n* = 4/14) of participants for the CAB LA injection and 21% (*n* = 3/14) for the RPV LA injection.

## DISCUSSION

CAB + RPV LA is currently administered monthly or every 2 months via IM injections into the ventrogluteal (recommended) or dorsogluteal muscle (4). The results of this study show that the lateral thigh muscle could be a potential alternative site of administration for CAB + RPV LA, helping to alleviate injection site fatigue or intolerability. The lateral thigh muscle could also be used in individuals with inaccessibility of the gluteal muscle. Notably, this study exceeded its target enrollment of 30% female participants.

The observed geometric mean and median CAB and RPV trough concentrations following lateral thigh dosing remained above their respective PA-IC_90_ values (CAB, 0.166 µg/mL; RPV, 12.0 ng/mL) from Day 2 until at least Week 12. Geometric mean plasma CAB concentration following single IM doses administered in the lateral thigh at Week 4 was higher in this study (2.56 µg/mL) than the observed trough concentrations 4 weeks after initial 600 mg IM gluteal injections in participants living with HIV-1 in the Phase 3 FLAIR study (1.56 µg/mL) (9), as well as in uninfected healthy adults in the Phase 2 PrEP Study HPTN077 (males, 1.79 µg/mL; females, 1.33 µg/mL) (20). The observed geometric mean CAB t_½_, which reflects the absorption phase, following thigh administration of 21 days was comparable to that observed after gluteal administration in the 114433 Phase 1 study (geometric mean across doses: 25–54 days) (12). It was also shorter than estimates in HPTN077 (males, 45.3 days; females, 60.4 days) and shorter than predicted by population PK modeling [males, 5.6 weeks (39.2 days); females, 11.5 weeks (80.5 days)] (10, 14). Finally, geometric mean [coefficient of variation (CVb)] plasma exposures of CAB [C_max_, 3.38 µg/mL (66% CVb); AUC_0–Week 12_, 3,202 hours × μg/mL (32.8% CVb)] following 600 mg IM injections were comparable to those observed after the first injection in the 114433 Phase 1 study, in which healthy adults were administered a single dose of CAB LA 800 mg via gluteal IM injections (split injections) [C_max_, 3.3 µg/mL (75.1% CVb); AUC_0–Week 12_, 3,851 hours × μg/mL (50.8% CVb)] and higher than the geometric mean CAB concentration observed 1 week following CAB LA 600 mg (start with injections, 1.89 µg/mL) (4, 12). The T_max_ of 7 days following IM thigh injection was consistent with the observed CAB T_max_ in historical IM gluteal studies (13, 21, 22). Relatively higher CAB Week 4 concentration and C_max_, as well as shorter t_½_, are suggestive of potential faster absorption following CAB LA administration to the lateral thigh as compared with the gluteus muscle. Differences are not likely attributable to HIV status, which is not a significant covariate in the CAB population PK model (14).

The geometric mean plasma RPV concentration following single IM doses administered in the lateral thigh at Week 4 was higher in this study in healthy adults (56.7 ng/mL) than the observed trough concentrations 4 weeks after initial 900 mg IM gluteal injections in the Phase 3 FLAIR study in participants living with HIV-1 (41.2 ng/mL) (9). The RPV PK profile after IM injections in the thigh was similar to that after gluteal IM injections in historical studies (8, 15). The T_max_ of 5 days for RPV administered via IM injection into the lateral thigh was within the range previously observed with gluteal injections of RPV LA 300 mg, 600 mg, and 900 mg in healthy volunteers (11.5, 9, and 3 days, respectively) (8). The geometric mean (CVb) C_max_ of 93.5 ng/mL (37.7%) observed in this study is also in line with those observed across doses for single gluteal injections [mean (SD): 300 mg, 39 ng/mL (25); 600 mg, 48 ng/mL (13); 1,200 mg, 140 ng/mL (16)] and in line with the geometric mean C_max_ after the first 900 mg RPV LA injection in participants with HIV-1 (direct to inject, 68 ng/mL) (4, 8). Geometric mean RPV plasma concentrations following a single IM dose of 900 mg administered to the lateral thigh were still detectable at Week 52. The geometric mean apparent t_½_ for RPV LA in this study was 137 days, which is shorter than the half-life of RPV after IM gluteal administration of 200 days, estimated based on population PK modeling using data collected across studies in healthy volunteers and participants with HIV-1 (15). However, it should be noted that RPV LA t_½_ could not be assessed accurately within the 52-week time frame for several participants, which could have also contributed to the relatively short observed apparent half-life in this study.

The safety and tolerability profiles observed with CAB LA and RPV LA IM injections to the lateral thigh were broadly similar to those observed with initial gluteal injections in previous studies, with no new safety signals identified (6, 9, 23). The most frequently reported AEs related to study drugs were ISRs, reported in all participants (100%) who received an injection. Comparable results were observed across the Phase 3/3b program of gluteal CAB + RPV LA, in which most participants (up to 84%) reported ISRs with their initiation gluteal injection (6, 7, 9). Injection site pain was the most commonly reported ISR, as previously reported across Phase 2/3/3b studies (6, 7, 9, 11, 24). Injection site erythema, induration, bruising, warmth, and pruritus were also reported. ISRs were almost all Grade 1 or 2 (94%) in this study. This is aligned with the results of a pooled ISR analysis from the FLAIR and ATLAS-2M studies, in which 94–97% of participants experiencing ISRs after their initiation gluteal injections had maximum Grade 1 or 2 events (25). In the Phase 3 program to date, withdrawals due to ISRs were an infrequent cause of discontinuation (6, 17, 26).

The results of the NRS for tolerability of injections showed that pain was highest at Day 2 post injection for both CAB LA and RPV LA. Scores decreased over time, in alignment with the short ISR duration (median, ~3 days) seen in the Phase 3/3b program for gluteal CAB + RPV LA injections (6, 7, 9).

Implications of the results from the current analysis are limited by the small sample size. Therefore, correlations between PK parameters and demographics could not be adequately evaluated. Interpretation may also be affected by the limited 24.3–34.4 kg/m^2^ range of BMI represented in the study, and individuals with BMI values outside of this range may have differing muscle capacity to receive thigh IM injections. Furthermore, it should be noted that the safety and tolerability profiles in healthy volunteers are compared with historical studies in participants living with HIV-1. Thigh IM injections are being further evaluated in a substudy of the ongoing Phase 3b ATLAS-2M study, in a population of participants living with HIV-1 who have received gluteal injections for more than 3 years, in order to establish the potential for use in the target population (27). Sampling duration in the present study may have limited the accuracy for the estimates of AUC_0–∞_, t_½_, and KA_LA_ for RPV LA, which may have been improved with more than 1 year follow-up.

### Conclusions

CAB and RPV PK data following a single IM injection into the lateral thigh muscle supported further evaluation of thigh IM administration in target populations of people living with HIV-1. The safety profiles of CAB LA and RPV LA IM injections to the lateral thigh muscle of healthy adult participants were comparable to the known safety profiles for CAB LA and RPV LA IM gluteal injections.

## SUPPLEMENTAL MATERIAL

Supplemental file 1 aac.00781-23-s0001.docx Fig. S1 to S4 and Table S1

ASM does not own the copyrights to Supplemental Material that may be linked to, or accessed through, an article. The authors have granted ASM a non-exclusive, world-wide license to publish the Supplemental Material files. Please contact the corresponding author directly for reuse.
