# Supplementary material for: Pharmacokinetics and tolerability of cabotegravir and rilpivirine long-acting intramuscular injections to the vastus lateralis (lateral thigh) muscles of healthy adult participants
Source: Antimicrob Agents Chemother. 2023 Dec 1;68(1):e00781-23. doi: 10.1128/aac.00781-23 (PMC10777827; doi:10.1128/aac.00781-23)
Supplement: Supplemental file 1 — Fig. S1 to S4 and Table S1 [file aac.00781-23-s0001.docx]

Supplementary Figure 1. CAB and RPV PK for pregnancy case 2


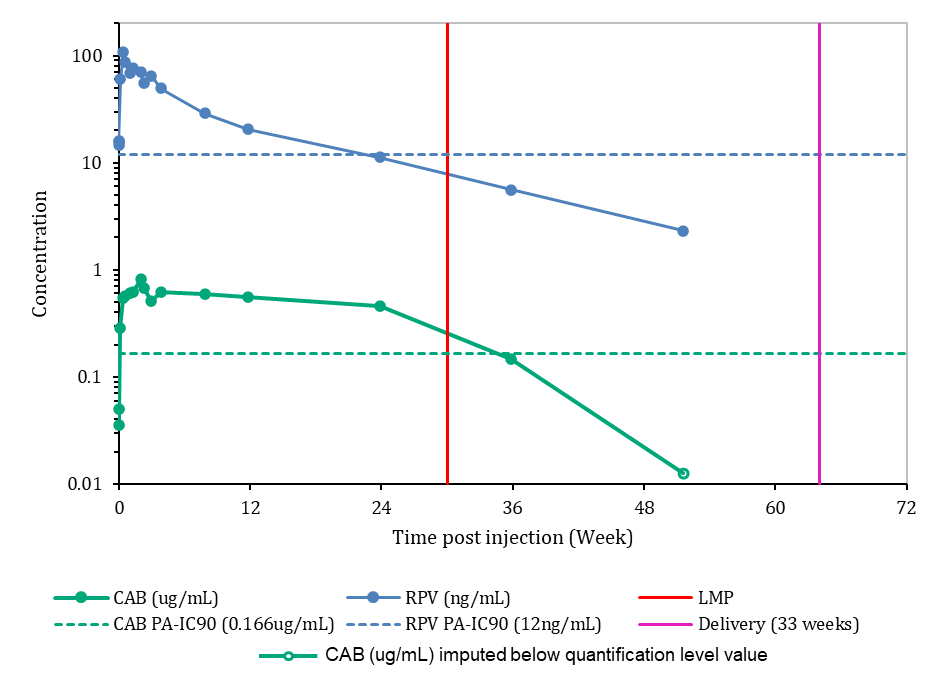

CAB, cabotegravir; LMP, last menstrual period; PA-IC_90_, protein-adjusted 90% inhibitory concentration; PK, pharmacokinetic; RPV, rilpivirine.

Supplementary Figure 2. Tolerability of injections (NRS)

*Scored from 0 “no pain” to 10 “extreme pain.”
CAB, cabotegravir; IM, intramuscular; NRS, Numerical Rating Scale; RPV, rilpivirine; SD, standard deviation.

Supplementary Figure 3. Perception of Injection (PIN domain scores)

*Scored from 1 “totally acceptable” to 5 “not at all acceptable.”
CAB, cabotegravir; IM, intramuscular; ISR, injection site reaction; PIN, Perception of Injection;
RPV, rilpivirine; SD, standard deviation.

Supplementary Figure 4. Perception of Injection (PIN individual item scores)

*Scored from 1 “totally acceptable” to 5 “not at all acceptable.”
CAB, cabotegravir; IM, intramuscular; PIN, Perception of Injection; RPV, rilpivirine; SD, standard deviation.

Supplementary Table 1. ISR participant-level safety overview

| **Parameter** | **CAB + RPV LA  (N=15)** |
| --- | --- |
| Participants who received ≥1 injection, n (%) | 14 (93) |
| Participants with ISRs, n (% of participants with injections) | 14 (100) |
| Injection site pain | 14 (100) |
| Injection site erythema | 8 (57) |
| Injection site induration | 7 (50) |
| Injection site swelling | 6 (43) |
| Injection site bruising | 4 (29) |
| Injection site warmth | 3 (21) |
| Injection site pruritus | 2 (14) |
| Participants with Grade 3 ISRs (maximum grade), n (% of participants with injections)* | 3 (21) |
| Withdrawal for injection-related reasons, n | 0 |

*No Grade 4 or 5 ISRs were reported.
CAB, cabotegravir; ISR, injection site reaction; LA, long-acting; RPV, rilpivirine.
